# Regions of homozygosity confer a worse prognostic impact in myelodysplastic syndrome with normal karyotype

**DOI:** 10.1002/jha2.651

**Published:** 2023-02-07

**Authors:** Mar Mallo, Heinz Tuechler, Leonor Arenillas, Sophie Raynaud, Thomas Cluzeau, Lee‐Yung Shih, Chiang Tung‐Liang, Christina Ganster, Katayoon Shirneshan, Detlef Haase, Martí Mascaró, Laura Palomo, José Cervera, Esperanza Such, Nicola Trim, Sally Jeffries, Emma Ridgway, Giovanni Marconi, Giovanni Martinelli, Francesc Solé

**Affiliations:** ^1^ MDS Research Group Institut de Recerca Contra la Leucèmia Josep Carreras (IJC) ICO‐Hospital Germans Trias i Pujol Universitat Autònoma de Barcelona Badalona Spain; ^2^ Microarrays Unit Institut de Recerca Contra la Leucèmia Josep Carreras (IJC) ICO‐Hospital Germans Trias i Pujol Universitat Autònoma de Barcelona Badalona Spain; ^3^ Boltzmann Institute for Leukaemia Research and Hematology Vienna Austria; ^4^ Hematological Cytology Laboratory Pathology Department Hospital del Mar GRETNHE, IMIM (Hospital del Mar Research Institute) Barcelona Spain; ^5^ Hematology Department Cote d'Azur University CHU of Nice Nice France; ^6^ Division of Hematology Chang Gung Memorial Hospital‐Linkuo Chang Gung University Taoyuan City Taiwan; ^7^ Clinics of Hematology and Medical Oncology University Medical Center Göttingen Göttingen Germany; ^8^ Hematology Service Hospital Son Llàtzer Palma de Mallorca Spain; ^9^ Experimental Hematology Vall d'Hebron Institute of Oncology (VHIO) Vall d'Hebron Barcelona Hospital Campus Barcelona Spain; ^10^ Hematology Service Hospital Universitario La Fe Valencia Spain; ^11^ West Midlands Regional Genetics Laboratory Birmingham Women's Hospital Birmingham UK; ^12^ IRCCS Istituto Romagnolo per lo Studio dei Tumori (IRST) “Dino Amadori” Meldola Italy

**Keywords:** chromosome, cytogenetics, microarrays, molecular cytogenetics, myelodysplastic syndromes

## Abstract

Half of the myelodysplastic syndromes (MDS) have normal karyotype by conventional banding analysis. The percentage of true normal karyotype cases can be reduced by 20–30% with the complementary application of genomic microarrays. We here present a multicenter collaborative study of 163 MDS cases with a normal karyotype (≥10 metaphases) at diagnosis. All cases were analyzed with the ThermoFisher® microarray (either SNP 6.0 or CytoScan HD) for the identification of both copy number alteration(CNA) and regions of homozygosity (ROH). Our series supports that 25 Mb cut‐off as having the most prognostic impact, even after adjustment by IPSS‐R. This study highlights the importance of microarrays in MDS patients, to detect CNAs and especially to detect acquired ROH which has demonstrated a high prognostic impact.

## 1 INTRODUCTION

Myelodysplastic syndromes (MDS) constitute a group of heterogeneous disorders, whose current diagnosis requires integration from different disciplines. Cytogenetics is the current gold standard for cytogenetic diagnosis and it is one of the main prognostic factors in the revised international prognostic scoring system (IPSS‐R) [1]. The mutational profile has been shown to be an important prognostic factor and has been incorporated into the new molecular prognostic scoring system (IPSS‐M) [2]. Here, we report the prognosis role of microarrays in MDS with normal karyotype, especially for regions of homozygosity (ROH).

Cytogenetics includes several techniques: chromosome banding analysis (CBA), fluorescence in situ hybridization, genomic microarrays, and, more recently, optical genome mapping [3]. Of these, only chromosome‐level events that may be discovered with classical CBA are included as a prognostic parameter in the IPSS‐R [1] and it has been retained in the new IPSS‐M [2]. With traditional techniques, half of MDS cases are found to have a normal karyotype. In‐depth analyses, with advanced techniques, have shown that the percentage of true normal karyotype cases can be reduced by 20–30% by the complementary application of genomic microarrays [4, 5]. Thus, the addition of genomic microarrays can increase the diagnostic yield and correctly classify a substantial portion of MDS patients that may otherwise be misclassified with the standard approach of karyotyping alone.

## 2 MATERIALS AND METHODS

We here present a multicenter collaborative study of 163 MDS *de novo* cases with a normal karyotype (15 of them had more than 10 metaphases completely analyzed) at diagnosis. All cases were analyzed in parallel with the ThermoFisher® microarray (either SNP 6.0 or CytoScan HD) for the identification of both CNA and ROH. We collected microarray results information from different centers which were integrated into a common database for statistical evaluation. CNA and ROH results were submitted to the common database following their own's center criteria.

First, we performed descriptive analyses of demographic data. For the comparison of subgroups, we applied Chi‐square and Kruskal–Wallis tests. Overall survival (OS) was defined as the time from diagnosis to the last available follow‐up or death. Time to acute myeloid leukemia (AML) transformation was defined as the time from diagnosis to AML date. Statistical comparisons between different curves were based on log‐rank tests and were calculated for patients with available follow‐up data (*n* = 138).

## 3 RESULTS

**FIGURE 1:**
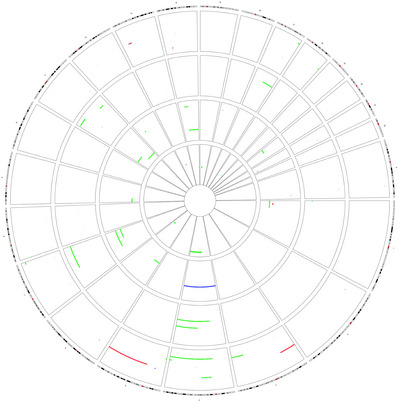
Circos plot displaying genetic aberrations found by microarrays. Blue bars indicate gains; red bars, losses; and green bars, regions of homozigosity.

According to SNP‐A analysis, 73/163 (44.8%) cases showed alterations (CNA and/or ROH). Table S1 summarizes patients’ characteristics. The median number of alterations per patient was 0 (range: 0–8). Considering altered cases, the median size aberration was 404 Kb (range: 24.1 Kb–190 Mb). Focusing on the type of alteration, 56 (34.4%) cases harbored CNA and 26 (15.95%) ROH. Considering the size of the affected genome in aberrant cases, the median value for CNA was 429 Kb (range: 69 Kb–190 Mb), for ROH, 25 Mb (range: 2–138 Mb); and for CNA plus ROH, 1 Mb (range: 69 Kb–190 Mb). Microarray results are given in detail in Figure 1.

Regarding clinical variables, no differences were observed between altered and normal cases, in terms of age, sex, blood counts, and IPSS‐R. OS and time to transformation were analyzed according to microarray results for 138 patients (Table 1).

**TABLE 1:** Prognostic impact of microarray alterations for both standard analysis and analysis adjusted by IPSS‐R

	Standard analysis (*n* = 138)				Analysis adjusted by IPSS‐R (*n* = 110)			
	Overall survival (mo)	*p* value	AML evolution (mo)	*p* value	Overall survival (mo)	*p* value	AML evolution (mo)	*p* value
**Normal versus altered**	59.73 versus 46.65	0.504	NR	0.778	53.59 versus 44.55	0.063	NR versus 65.22	0.407
**Harboring ROH alone (no vs. yes)**	64.69 versus 19.19	0.054	NR	0.336	45.54 versus 19.19	0.098	NR versus 65.22	0.265
**ROH (<25 vs. >25 Mb)**	64.69 versus 17.71	0.001*	NR	0.033*	46.62 versus 19.19	0.032*	NR	0.067

**Abbreviations**: CNA, copy number alteration; mo, months; NR, not reached; ROH, region of homozygosity.

* Statistically significant.

**FIGURE 2:**
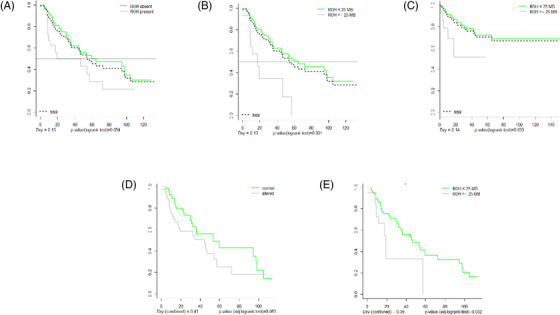
(A) OS curve for the presence or absence of ROH alterations. (B) OS curve considering the presence of ROH greater than 25 Mb. (C) AML evolution considering the presence of ROH greater than 25 Mb. (D) OS curve, after IPSS‐R adjustment, for microarray result (normal vs. altered). (E) OS curve, after IPSS‐R adjustment, considering the presence of ROH greater than 25 Mb. Abbreviations: AML, acute myeloid leukemia; IPSS‐R, revised international prognostic scoring system; OS, overall survival; ROH, region of homozygosity.

A positive microarray result indicating ROH showed a borderline significant negative impact on OS (Figure 2A), while categorization of ROH in up to 25 Mb versus more than 25 Mb showed a strong and significant negative impact of ROH on both OS and AML evolution (Figure 2B,C). To compensate for differing risk distributions, we performed analyses adjusting for IPSS‐R in 110 patients (Table 1). Microarray results in general (considering CNA and ROH) only show a tendency for a lower OS in patients with a positive abnormal microarray result (*p* = 0.063) (Figure 2D). The presence of ROH greater than 25 Mb, however, maintains its prognostic impact for OS (*p* = 0.032) (Figure 2E).

## 4 DISCUSSION

In the next‐generation sequencing era, microarrays are still useful for routine clinical investigation. Several studies have shown their successful applicability in MDS, especially in cases with a low number of metaphases and/or unsuccessful cytogenetic studies [6, 7]. The first studies appeared in 2009 [8] and showed the utility of germline control samples, especially for ROH. However, this approach is not always feasible because the costs per analysis are doubled (normal plus tumor DNA). Maciejewski et al. established a cut‐off of 25 Mb to distinguish germline from acquired ROH [9]. Our series supports that 25 Mb cut‐off as having the most prognostic impact, even after adjustment by IPSS‐R. The prognostic impact of ROH was also stated by Palomo et al. in a series of 128 chronic myelomonocytic leukemia with normal karyotype [10]. One of the largest studies in MDS was published in 2011 by Tiu et al. [4], with 430 myeloid patients (118 MDS patients with a normal karyotype). In 2016, Volkert et al. [5] presented the largest series so far, of 520 MDS with normal karyotype. However, this study was performed with Array Comparative Genomic Hybridization (aCGH), which is unable to detect ROH. Compared to our series, the detection rate of abnormalities was rather low, with 11% of cases only. The microarray used was 270K aCGH, whereas our series was performed with SNP arrays that includes more than 1.8 million probes, combining copy number and SNP probes. We postulate that the difference in the incidence of alterations (11% vs. 45%) could be due to the difference in the resolution of arrays used. The range of alterations was 174 Kb–3.4 Mb for Volkert et al., and 24 Kb–190 Mb for our series. However, assessing the prognostic impact of small alterations is difficult because of the lack of recurrent aberrations. Volkert et al. highlighted the prognostic impact of deletions [5], but we were not able to confirm their prognostic impact in our cohort, in which the main adverse prognostic factor in our cohort was the presence of ROH greater than 25 Mb.

Microarray guidelines are essential for diagnostic applications [11]. In 2018, Kanagal‐Shamanna et al. published a review article focused on the clinical utility of microarrays, pointing out the utility of ROH detection [12]. Although molecular studies are becoming more and more essential for MDS diagnosis and stratification [2], SNP microarrays are valuable complementary techniques. An AML study using SNP microarrays showed that 50–100% of cases harboring deletions or ROH, in distinct myeloid genes, show point mutations of the same genes [13]. Detection of CNA and ROH is important, especially to determine the *TP53* status in MDS. Pathogenic TP53 alterations (point or indel mutations and 17p deletions and/or ROH) are detected in 7–11% of MDS. About two‐thirds have multiple mutation hits (multi‐hit), consistent with biallelic TP53 alterations. Of these, more than 90% of TP53 mutated patients have complex or very complex karyotypes and they can be addressed as AML‐equivalent for therapy considerations [2, 14, 15].

This study highlights the importance of microarrays in MDS patients, to detect CNAs and especially to detect acquired ROH which has demonstrated a high prognostic impact.

## CONFLICT OF INTEREST STATEMENT

The authors declare they have no conflicts of interest.

## Supporting information

Supporting Information.
