# Supplementary material for: Regions of homozygosity confer a worse prognostic impact in myelodysplastic syndrome with normal karyotype
Source: EJHaem. 2023 Feb 7;4(2):446–9. doi: 10.1002/jha2.651 (PMC10188467; doi:10.1002/jha2.651)
Supplement: Supplementary file 1 — Supporting Information. [file JHA2-4-446-s001.docx]

**Supplementary Table 1. Description of the series**

|  | **Number of patients, n(%)** | **Median** |
| --- | --- | --- |
| **Total** | 163 (100) | - |
| **Age** | 163 (100) | 73.2 yr |
| **Sex** | | |
| Male | 91 (55.8) | - |
| Female | 72 (44.2) | - |
| **Haemoglobin** | 118 (72.4) | 10g/dL |
| **Neutrophil** | 104 (63.8) | 48.8% |
| **Platelet count** | 118 (72.4) | 136*10^9^/L |
| **Bone marrow blast count** | 143 (87.7) | 2% |
| **IPSS-R** | | |
| Very low | 24 (14.7) | - |
| Low | 55 (33.7) | - |
| Intermediate | 30 (18.4) | - |
| High | 11 (6.7) | - |
| Very high | 6 (3.7) | - |
| Unknown | 37 (22.7) | - |
| **Microarray result** | | |
| Normal | 90 (55.21) | - |
| Altered | 73 (44.79) | - |
| 1 alteration | 47 (64.4) | - |
| >1 alteration | 26 (35.6) | - |
